# Scotland's 2009–2015 methadone-prescription cohort: Quintiles for daily dose of prescribed methadone and risk of methadone-specific death

**DOI:** 10.1111/bcp.14432

**Published:** 2020-07-06

**Authors:** Lu Gao, J. Roy Robertson, Sheila M. Bird

**Affiliations:** 1 MRC Biostatistics Unit, University of Cambridge School of Clinical Medicine, Cambridge, UK; 2 University of Edinburgh Usher Institute, Edinburgh, UK; 3 University of Edinburgh Centre for Medical Informatics, Edinburgh, UK

**Keywords:** age group, female, hazards for sex, age group and top quintile for daily dose of methadone, methadone-prescription clients, methadone-specific deaths, recovery rules for prescribed daily dose

## Abstract

**Aims:**

As methadone clients age, their drug-related death (DRD) risks increase, more than doubling at 45+ years for methadone-specific DRDs.

**Methods:**

Using Community Health Index (CHI) numbers, mortality to 31 December 2015 was ascertained for 36 347 methadone-prescription clients in Scotland during 2009–2015. Cohort entry, quantity of prescribed methadone and daily dose (actual or recovered by effective, simple rules) were defined by clients' first CHI-identified methadone prescription after 30 June 2009 and used in proportional hazards analysis. As custodian of death records, National Records of Scotland identified non-DRDs from DRDs. Methadone-specific DRD means methadone was implicated but neither heroin nor buprenorphine.

**Results:**

The cohort's 192 928 person-years included 1857 non-DRDs and 1323 DRDs (42%), 546 of which were methadone specific. Actual/recovered daily dose was available for 26 533 (73%) clients who experienced 420 methadone-specific DRDs. Top quintile for daily dose at first CHI-identified methadone prescription was >90 mg. Age 45+ years at cohort-entry (hazard ratio vs 25–34 years: 3.1, 95% confidence interval: 2.4–4.2), top quintile for baseline daily dose of prescribed methadone (vs 50–70 mg: 1.9, 1.1–3.1) and being female (1.3, 1.0–1.6) significantly increased clients' risk of methadone-specific DRD.

**Conclusion:**

Extra care is needed when methadone daily dose exceeds 90 mg. Females' higher risk for methadone-specific DRD is new and needs validation. Further analyses of prescribed daily dose linked to mortality for large cohorts of methadone clients are needed internationally, together with greater pharmacodynamic and pharmacokinetic understanding of methadone by age and sex. Balancing age-related risks is challenging for prescribers who manage chronic opiate dependency against additional uncertainty about the nature, strength and pharmacological characteristics of drugs from illegal markets.

## 1 Introduction

By the 1990s, UK's heroin injector epidemics of the early 1980s were being countered by opioid substitution therapy, primarily methadone, which not only reduced drug-related deaths (DRDs)^1,2^but also blood-borne virus transmissions and criminality.^3,4^ Scotland's methadone-prescription cohort^5^includes many who are former injectors, of whom at least half will be hepatitis C virus carriers^6,7^; most smoke, and misuse of alcohol, psychiatric and physical comorbidities are also not uncommon.^8,9^

Despite the remarkable fall in UK's DRD-rate per 1 million defined daily doses of methadone in the early 21^st^ century,^1^ the present decade has seen sharply increased numbers of opioid-related deaths.^10^ The UK's increase was anticipated by evidence syntheses^11–13^ and by national record-linkage studies of virtual cohorts of opioid-dependent clients.^3,14^ Both forewarned about demographic influences (sex, age group) on DRD rates, including a strong sex by age-group interaction in DRD-risk for opioid users.^5,14^ Discovering that females' advantage, in terms of lower DRD-risk, diminished with age^14^ is important for risk prediction^15^ but was unrecognized in early systematic reviews.^16^

We became concerned about a possible role for prescribed methadone in Scotland's rise in methadone deaths in the second decade of the 21^st^ century.^5^ Indeed, Scotland's 2009–2013 methadone-prescription cohort demonstrated that clients' risk of methadonespecific DRD increased more strikingly with age than for all DRDs.^5^ Quickly, the cohort of opioid users in England's 2005–09 National Drug Treatment Management System was used to validate the Scottish results.^17^ This English record-linkage cohort could adjust for a triad of major behavioural risk factors (injector status, misuse of alcohol, misuse of benzodiazepines) but lacked information on the prescribed quantity or daily dose of methadone. Synthesis of the 2 UK studies suggested that the risk of methadone-specific DRD tripled by age 45+ years (95% confidence interval [CI]: 3.0 to 4.7) compared with 25–34 years.^17^

Meanwhile, based on 87 DRDs in a primary care cohort, Hickman et al.^18^ considered confounding between the choice of opioid substitution therapy (methadone vs buprenorphine) and the client's age group or number of comorbidities, both of which are potentially implicated in methadone-specific DRDs.^5,17,19–22^

Internationally, there is a dearth of information on the daily dose of prescribed methadone for large cohorts of opioid-dependent clients.^15,16^

Our aim is to provide alternative proportional hazards (PH) analysis for methadone-specific DRDs in Scotland's 2009–2015 methadone-prescription cohort based on:

- clients' sex, age group at accrual, prescription source and baseline quintile for quantity (qQs) of prescribed methadone; or
- clients' sex, age group at accrual, prescription source and baseline quintile for daily dose (dQs) of prescribed methadone.

## 2 Methods

### 2.1 Definitions: DRDs

We applied the UK harmonized definition of DRD.^23^ National Records of Scotland^10^ provided information on the opioid specificity of Scotland's DRDs: methadone-specific DRDs: methadone was implicated in DRD but neither heroin/morphine nor buprenorphine implicated; heroin-specific DRDs: heroin/morphine was implicated in DRD but neither methadone nor buprenorphine implicated; and heroin– methadone DRDs: methadone and heroin/morphine both implicated in DRD but buprenorphine was not implicated.

In appraising which drugs are implicated as causal factors in any DRD and which, although present, probably did not contribute, Scotland's pathologists are supported by having a national protocol for toxicological testing at forensic autopsies.

### 2.2 Scotland's Community Health Index

Scotland's Community Health Index (CHI) is a register of all patients in National Health Service (NHS) Scotland, Scotland's publicly funded healthcare system. From birth, patients are identified by a 10-digit CHI number, usually the patient's date of birth (DDMMYY) followed by 4 digits: 2 randomly generated, the third identifying sex (odd for males), and the fourth a check digit. The CHI numbers are key to Scotland's trusted record linkage,^24^ not least because deaths and hospitalizations are CHI identified.

### 2.3 Scotland's methadone-prescription client cohort for 2009–2015: data sources and linkage

**Figure 1:**
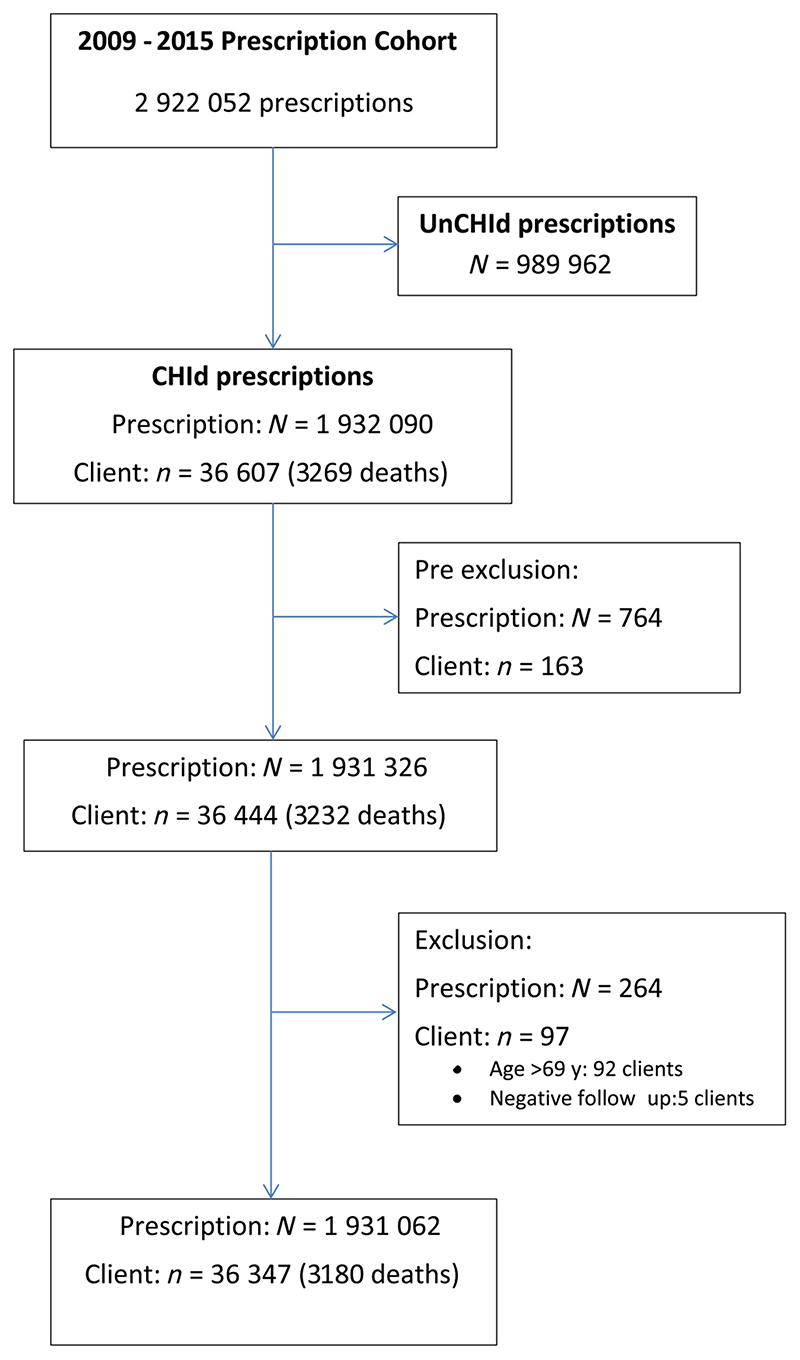
Exploratory data analysis: from Scotland's >2.9 million methadone prescriptions during 1 July 2009 to 30 June 2015 to 36 347 Community Health Index (CHI)-identified methadone-prescription clients followed-up to 31 December 2015

Methadone prescriptions for opioid substitution therapy fall within a specific classification category in Scotland's National Prescribing Information System^25^: most are CHI identified, see Figure 1. All give quantity of methadone prescribed and number of instalments by which the prescribed quantity is issued, as both are required for the reimbursement of pharmacists. Daily dose of prescribed methadone is not routinely available in electronic format.^5,26^ But daily dose of prescribed methadone is available electronically for a subset of general practitioner (GP) prescriptions; and was extracted by Scotland's Information Services Division using Natural Language Programs applied to GPs' electronic messaging.^26^

Instalment dispensing is different in Scotland: on regular GP prescriptions, any drug is allowed and can be for any duration, although good practice suggests a limit of 28 days. In England, instalment prescriptions are for a maximum of 14 days and only allow Schedule 2 Controlled Drugs under the Misuse of Drugs Act 1971. England's 14-day limit means that quantities of prescribed methadone as large as in Scotland are unlikely.

To define Scotland's methadone-prescription client cohort for 2009–2015, nearly 3 million methadone prescriptions during 1 July 2009 to 30 June 2015 were assessed for linkage to Scotland's mortality records to 31 December 2015. As all deaths are CHI identified, prescriptions' CHI number was used for this exact linkage. The CHI number was also used to link serial CHI-identified methadone prescriptions for the same client.

For CHI-indexed methadone prescriptions, we obtained: client's sex, age in completed years at 1 July of prescription year (enabling current age group to be used in a sensitivity analysis); prescription date (when missing, the later reimbursement date was used^5^); reimbursement date; prescription source (GP; other source); quantity of prescribed methadone per prescription; number of instalments per prescription; daily dose of prescribed methadone (if extractable by Natural Language Program from GPs' electronic messaging^5,26^); full date of death; whether underlying cause of death was non-DRD or DRD; and DRDs' opioid specificity.

We defined Scotland's 2009–2015 methadone-prescription client cohort as: clients with 1 or more CHI-identified methadone prescription during 1 July 2009 to 30 June 2015. The client's first CHI-identified methadone prescription during 1 July 2009 to 30 June 2015 defined cohort entry or accrual date, baseline quantity (also daily dose) of prescribed methadone and age group at accrual (<25, 25–34, 35–44, 45+ years).

As full date of death is potentially identifying, approval by Scotland's Public Benefit and Privacy Panel for this study required that all computations were within the Usher Institute safe haven.

### 2.4 Exclusion criteria

Four types of data checking were undertaken. First, survival time from the date of CHI-identified client's baseline methadone prescription was computed: negative survival times were checked for evidence of incorrect linkage. Secondly, wide outer bounds were defined for 4 variables, see below: CHI-identified prescriptions falling outside of any of these outer bounds were excluded as widely implausible. Third, as before^5^ and on account of substitution of reimbursement date for missing prescription dates, we added 60 days to all CHI-identified survival intervals to ensure positivity: any residual negative times resulted in client exclusion. Finally, a plausible upper bound of 69 years was set for age at cohort entry for clients receiving methadone substitution: breach of this upper bound resulted in client exclusion.

#### 2.4.1 Incorrect linkage

Computing time in days from the date of the client's baseline CHI-identified methadone prescription to the earlier of death date or 31 December 2015, identified 63 negative survival times. On cross checking, Information Services Division confirmed only 7 (hereafter deleted) were linked incorrectly.

#### 2.4.2 Wide outer bounds

CHI-identified methadone prescriptions in 2009–2015 were excluded if any of the following applied on a per-prescription basis:

- quantity prescribed: <5 mg or >12 000 mg;
- instalments: <1 or >84;
- daily dose: <1 mg or >300 mg;
- age-in-prescription-year: <5 years or >79 years.

The analysis file was thereby reduced by 764 CHI-identified methadone prescriptions (0.04%) to 1 931 326; clients by 163 to 36 444; deaths by 37, see Figure 1.

#### 2.4.3 Ensuring positivity

As in an earlier analysis,^5^ because reimbursement date was substituted for missing prescription date, 60 days were added to all survival times. Positivity was assured for all except for 5 CHI-identified clients, who were excluded.

#### 2.4.4 Plausible upper bound for age at cohort entry

Ninety-two CHI-identified clients were excluded because age at cohort entry was >69 years.

The 2 checks above together excluded 97 CHI-identified clients: 52/97 had died; 2/52 were DRDs. There remained 36 347 CHI-identified methadone-prescription clients.

### 2.5 Simple rules for establishing daily dose: Derivation and validation

For this paper, we devised and verified simple rules for recovery of daily dose from quantity of prescribed methadone and number of instalments, see Appendix 1.

Simple rules (hereafter, recovery rules) were devised for establishing daily dose from quantity of methadone prescribed and number of instalments at first CHI-identified prescription. Not every instalment number had an acceptable rule, see below. Recovery rules were derived for CHI-identified clients who had most recently joined the cohort so that our derivation subcohort comprised:

- clients from the 2009–2013 cohort^5^ who received 1 or more CHI-identified methadone prescription after 30 June 2013 and were alive after 31 December 2013—their accrual date to the derivation subcohort was the later of 1 January 2014 and date of their subcohort-qualifying first CHI-identified methadone prescription after 30 June 2013; and
- new clients, not part of the 2009–2013 cohort,^5^ who received 1 or more CHI-identified methadone prescriptions after 30 June 2013 and whose accrual date was the date of their qualifying first CHI-identified methadone prescription after 30 June 2013.

Conditional on number of instalments, a recovery rule would be accepted if it correctly recovered at least 75% of the actual daily doses in clients' accrual month within the derivation subcohort. Our performance criteria were met when number of instalments for issuing the prescription was 4, 7, 14, 21, 28, 35, 42 or 56 (Appendix 1); and verified for Scotland's 2009–2015 methadone-prescription cohort. For these listed instalment numbers, excepting 4 and 7, performance criteria were met by simply dividing quantity by number of instalments; the recovery rules for 4 and 7 have an additional check step. All accepted rules were then applied to recover daily dose at cohort entry for CHI-identified clients.

### 2.6 Key covariates, including quintiles for daily dose of prescribed methadone

Key covariates were: prescription source (GP vs other prescriber), sex (female vs male) and age group at cohort-entry (<25 years, 25–34, 35–44, 45+ years). In addition,^5^ quintile for quantity of prescribed methadone at first CHI-identified prescription (qQ) was available for all clients.

For 26 533 (73%) clients, recovery rules allowed quintile for daily dose of prescribed methadone at first CHI-identified prescription (dQ) to be analysed. Quintiles partition clients into fifths according to prescribed daily dose: from the 20% receiving the lowest fifth of baseline prescribed daily doses (dQ1) to the top 20% of prescribed daily doses of methadone (dQ5). Using quintile indicators allows the association between hazard ratio (HR) and increasing baseline prescribed daily dose to be made explicit in PH regression analysis.

### 2.7 Statistical analysis

After documenting the impact of exclusion criteria on prescriptions, clients and deaths, we summarize the performance of recovery rules for daily dose for Scotland's 2009–2015 methadone-prescription cohort.

Next, for each covariate level, we provide DRD rates and methadone-specific DRD rates for the cohort as a whole; and when restricted to clients with actual or recovered daily dose at first CHI-identified prescription.

Using PH regression analysis, we assess how steeply HRs increase by age group at accrual for methadone-specific DRDs; and how influential—based on regression χ^2^ values on 4 degrees of freedom— are dQs vs qQs. Adjusted HRs and 95% CIs are estimated simultaneously, relative to each covariate's baseline category as shown in tables. Appendix 2 includes corresponding PH analyses for all DRDs which, unlike methadone-specific DRDs, require sex by age group interaction to be taken into account. All analyses were performed using STATA v15.1; STATA's stcox was used for PH analysis.

### 2.8 Sensitivity analysis

Appendix 3 includes 3 types of sensitivity analysis. First, rather than age group at cohort entry, we fitted time-updated age group since a single transition to an older age group could have occurred. The second analysis focuses on GP clients solely as GP prescriptions alone were the basis for our recovery rules. Thirdly, the key PH analysis for methadone-specific DRDs that incorporated dose quintiles was repeated separately for: (i) CHI-identified clients who entered the Scotland's 2009–2015 methadone-prescription cohort during July to December 2009 (mainly as prevalent clients); and (ii) CHI-identified clients who entered the cohort during 2010 and 30 June 2015 (including relatively more incident clients in their methadone titration phase).

## 3 Results

### 3.1 Exploratory data analysis

#### 3.1.1 Exclusions

After exclusion steps (see Figure 1), the 5^th^ and 95^th^ percentiles for all CHI-identified methadone prescriptions were: for quantity, 140 and 2970 mg, mean of 1221 mg (standard deviation [SD] 1025); for number of instalments, 1 and 84, mean of 13.3 instalments (SD 9.6); for actual daily dose, 16 and 120 mg, mean of 64 mg (SD 34)— available for only 736 153 (38%) of all CHI-identified prescriptions, but for 51% of 1 429 863 CHI-identified GP prescriptions.

#### 3.1.2 Recovery rules and descriptive statistics

Eight accepted recovery rules for daily dose at cohort entry were generally of the form quantity prescribed divided by D(i) where the value for divisor D(i) depended on (i), the number of instalments (4, 7, 14, 21, 28, 35, 42 or 56), with extra conditions needed only when the number of instalments was 4 or 7 in order for at least 75% of actual daily doses to be recovered correctly, see Appendix 1: actual agreement rate was 88% overall.

Recovered or actual daily dose at cohort entry was available for 26 533 (73%) of clients in Scotland's 2009–2015 cohort, including for 7349 (58%) of the cohort's 12 743 CHI-identified clients whose prescriber was other source, see Table 1. Recovered or actual daily dose was available for 19 184 (81%) of 23 604 CHI-identified clients whose prescriber was GP.

Death rates for Scotland's 2009–2015 methadone-prescription cohort; and when restricted to 26 533 clients with actual or recovered daily dose of prescribed methadone.

Scotland's 2009–2015 methadone-prescription cohort comprised 36 347 CHI-identified methadone-prescription clients who experienced 1857 non-DRDs and 1323 DRDs, including 546 methadone-specific DRDs, in 192 928 person-years (py) of follow up, see Table 2.

Clients' non-DRD rate was 9.6 per 1,000 py vs their DRD rate of 6.9 per 1,000 py, both precisely estimated. The 65% of clients with a GP prescriber had lower DRD rate (and lower methadone-specific DRD rate) than clients whose prescriber was other source. Two-thirds of clients were male, for whom methadone-specific DRD rate was lower at 2.6 per 1000 py (95% CI: 2.4–2.9) than for females (3.2, 95% CI: 2.8–3.7).

Both DRD rate and methadone-specific DRD rate increased with age group at cohort entry, the latter more steeply. The modal age group at accrual to Scotland's 2009–2015 methadone-prescription cohort was 25–34 years (44% of clients) with only 8% of clients aged 15–24 years. Methadone-specific DRD rate was significantly higher for clients in the top quintile for prescribed quantity at accrual to the cohort (qQ5: 4.3 per 1000 py, 95% CI: 3.7–5.0) than for clients in the middle quintile (qQ3: 2.1 per 1000 py, 95% CI: 1.7–2.6).

Table 3 presents corresponding information for the 26 533 clients with actual or recovered daily dose of prescribed methadone at first CHI-identified prescription, 72% of whom had a GP prescriber. Their DRDs numbered 995, including 420 methadonespecific DRDs. Methadone-specific DRD rate was significantly higher for clients in the top quintile for prescribed daily dose at accrual (dQ5 [>90 mg]: 5.0 per 1000 py, 95% CI: 4.2–6.0) than for clients in the middle quintile (dQ3 [50–70 mg]: 2.7 per 1000 py, 95% CI: 2.2–3.3).

#### 3.1.3 Adjusted HRs for methadone-specific DRDs in Scotland's 2009–2015 methadone-prescription cohort

##### Baseline quintile for prescribed quantity (Table 4)

For methadone-specific DRDs, interaction between sex and agegroup is unnecessary (χ^2^ on 3 degrees of freedom of 4.00, P = .026, see Appendix 2). Females have higher HR (1.4) than males; HRs increase very steeply with age group at cohort entry, being 3-fold higher for clients 45+ years and 2-fold greater for clients aged 35–44 years than if 25–34 years old at cohort entry. Only the top quintile for quantity of prescribed methadone was associated with a significantly increased HR compared to qQ3. Clients whose prescription source was non-GP had significantly higher methadone-specific risk (HR, 1.36); likewise, DRD risk (see Appendix 2: HR, 1.31).

##### Baseline quintile for recovered or actual daily dose (Table 5)

For methadone-specific DRDs, females are disadvantaged (HR 1.3, 95% CI: 1.0–1.6) and the steepness of increase in HRs, both age related and by quintile for baseline daily dose, is much greater than for all DRDs (see Appendix 2). By age group at cohort entry, HR was 3-fold higher (95% CI: 2.4 to 4.2) at 45+ years than at 25-34 years. For dQ5, HR was also 3-fold higher (95% CI: 2.2 to 4.5) than for dQl and significantly greater than for dQ3 (HR = 3.15/1.68 or 1.88; 95% CI: 1.13 to 3.12), itself significantly greater than dQl. Finally, higher HR for methadone-specific DRD was again associated with other-source prescribers (HR, 1.3, 95% CI: 1.1–1.6).

Note that for 420 clients aged 45+ years at cohort entry who received baseline methadone daily dose >90 mg, methadone-specific DRD rate was 6.5 per 1000 py (95% CI: 3.9 to 10.7, based on 15 methadone-specific DRDs in 2138 py) vs 1.6 (95% CI: 1.1 to 2.4, based on 24 methadone-specific DRDs in 14 869 py) for 2627 clients in dQ3 and aged 25–34 years at cohort entry.

#### 3.1.4 Sensitivity analyses

See Appendix 3 for 3 sensitivity analyses. The first relates to current age group rather than age group at cohort entry (Table A4). Since clients' age group changes at most once during follow up, age effects sharpened only slightly with current age group as alternative to age group at accrual. The second focuses on GP clients only, as GP prescriptions were the basis for our recovery rules (Table A5), but still endorses Table 5. Thirdly, Appendix 3 re-estimates Table 5 for the subcohort of mainly prevalent CHI-identified clients whose cohort entry was in July to December 2009 (Table A6) vs later-recruited CHI-identified clients (Table A7). The mainly prevalent subcohort is the larger; clients are older at cohort entry, only 19% had a non-GP prescriber and only 5% had baseline daily dose in dQl. Nonetheless, inferences about age group and dQs are broadly similar for the 2 subcohorts. We note, however, that the mainly prevalent clients' hazard of methadone-specific DRD did not differ by prescription source.

## 4 Discussion

### 4.1 Summary of main findings

Daily dose at first CHI-identified methadone prescription was analysed for 73% of clients in Scotland's 2009-2015 methadone-prescription cohort. Daily doses >50 mg up to 70 mg comprised the mid-quintile (dQ3). Top quintile (dQ5) was daily dose >90 mg, within which mean daily dose (SD) was 117 mg (25).

For methadone-specific DRDs, HR increased steeply for the 2 older age groups and steadily with quintile for daily dose through to 3.1 (95% CI: 2.2 to 4.5) for dQ5; and females were at greater risk, a new finding.

For methadone-specific DRDs and for all DRDs, we found an increased HR (1.3) associated with non-GP prescribers: clients' physical and psychiatric comorbidities may be less well known by other prescribers than by GPs.

For context, non-DRDs outnumbered DRDs by 3:1 for methadone-prescription clients aged 45 years and over.

### 4.2 Key considerations

Notwithstanding the substantial reduction in harms (overdose deaths, criminality and blood-borne virus risks) that opioid substitution therapy has delivered for younger heroin users, the risk of methadonespecific DRD increases both as clients age into their 40s and 50s; and steadily with baseline quintile for daily dose of prescribed methadone.

Guidelines for methadone clients recommend a daily dose of 60–120 mg.^27^ Adherence to prior guidance was checked by prescribing surveys^28–31^ or evidence synthesis.^1^ However, Scotland's 2009–2015 methadone-prescription cohort is the first major cohort internationally to have analysed the joint effects of sex, age group and baseline daily dose of prescribed methadone on both methadonespecific DRDs and all DRDs.

In some individuals, females especially,^32^ methadone (unlike buprenorphine^18^) is associated with prolongation of the QTc interval leading to the development of Torsades de Pointes and cardiac arrest.^33^ Undiagnosed QTc prolongation may manifest as methadonespecific DRDs.

Periodic electrocardiograms are recommended for clients receiving >100 mg of methadone daily,^33^ but not achieved in practice. Other risk factors for QTc prolongation include comorbidities such as circulatory or liver disease; coprescribing for mental or physical ill health^34^; use of both methadone and cocaine; and being female.^32,35^ Could the latter partly explain our novel finding that females are at higher risk of methadone-specific DRD?^35–38^

Confounding between methadone-specific DRD risk and the client's daily dose of prescribed methadone (dQ) cannot be excluded. However, of Hill's 9 criteria for ascribing cause,^39^ dQs meet at least 6: strength of association, specificity (vs all DRDs), temporality, biological gradient, plausibility and coherence.

Balancing of prescribing risks is challenging in clinical practice, never more so than when managing chronic opiate dependency. Risks include^27^: the toxicity of prescribed opiates; coexisting respiratory, cardiovascular and metabolic hazards, for example from compromised liver capacity; the potential dangers of cumulative doses of multiple drugs beyond an individual patient's current tolerance; compounded by ignorance of the nature, strength and pharmacological characteristics of drugs that clients may access on the illegal market. The latter vary notoriously in time and place.

The above considerations produce an environment of high risk for overdose toxicity and sudden death. Information, as in this paper, which might mitigate some of these risks is, therefore, important.

We have raised a concern about higher DRD risks for methadone clients of non-GP prescribers: in particular, for methadone-prescription clients whose cohort entry date was during 2010–2015, a period when nonmedical prescribing in the management of substance misuse had expanded in Scotland, as elsewhere.^40^ Scotland's GPs have not been reluctant to manage methadone prescriptions for clients with major comorbidities as around 80% of CHI-identified clients whose cohort entry was July–December 2009 had GP prescribers. In terms of risk mitigation, it might be helpful for GP Summary Care Records to be routinely available to specialist service prescribers so that they are aware of ageing clients' comorbidities.

In a changing landscape of funding, commissioning of services and basic training of prescribers, both Scotland and the rest of the UK are moving towards a wider range of prescribers who include pharmacists and specialist nurses. Our paper draws attention to the complexity of need that ageing methadone clients have for interventions from a variety of chronic disease specialists (respiratory, cardiovascular and gastrointestinal) beyond their immediate drug problems. Primary care, in its widest sense, aspires to be the focus for multidisciplinary care.

### 4.3 Strengths and limitations of this study

First, Scotland has a national protocol for toxicology at forensic autopsies which underwrites the opioid-specificity of Scotland's DRDs. Second, and unparalleled for a national cohort, we could analyse quintiles for daily dose of prescribed methadone at cohortentry for over 26 500 methadone clients in 2009–2015 who experienced 995 DRDs, 420 of them methadone-specific DRDs. Third, representativeness in terms of daily dose at cohort entry is supported because actual or recovered daily dose was available for 73% of all clients in Scotland's CHI-identified methadone-client cohort; and for 81% of GP clients.

Fourth, to minimize ascertainment bias, we considered only the baseline (not time varying) quantity of methadone prescribed at first CHI-identified prescription. This first CHI-identified prescription defined the client's entry to Scotland's methadone-client cohort (2009–2015) but was not typically the client's first methadone prescription, especially if cohort entry was in 2009. Accrual date was July–December 2009 for 57% of clients, indicating that clients were mainly prevalent at cohort entry, see also Gao et al.^5^ Clients whose first CHI-identified methadone prescription occurred during 2010 to June 2015 include incident clients whose baseline daily dose of methadone was captured during the clients' titration phase.

Fifth, our results on quantity and daily dose were robust when restricted to GP clients only or based on current age group vs age group at cohort entry or when the mainly prevalent subcohort of clients whose cohort entry was in July to December 2009 was analysed separately.

There are several limitations. First, only 66% of all Scotland's methadone prescriptions in 2009–2015 were CHI-identified. However, as a best estimate, our 36 347 CHI-identified methadone clients represent 80% (plausible range: 70–90%) of Scotland's methadone clients during 2009–2015, because a substantial proportion of methadone prescriptions that lack a CHI number may pertain to already CHI-identified clients. Hence, we do not use time-updated quantity prescribed or daily dose because we cannot be certain that the most recent CHI-identified methadone prescription is the client's most recent methadone prescription.

In deriving simple rules for recovery of daily dose, a limitation was that actual daily doses were available electronically from GP prescriptions only but recovery rules were applied to other-source prescriptions on the reasonable assumption that the same relationships hold. As a check, PH analyses using quintiles for daily dose were repeated for GP-prescribed clients only, and inferences were essentially unaltered.

Record-linkage studies have limited scope for resolving data queries. We took a harder line than previously on exclusion criteria, respectively for prescriptions and clients.

The need to substitute the later reimbursement date for missing first prescription date was a minor issue. More importantly, we did not know, and so could not analyse, when clients exited from methadone therapy as the date of their last CHI-identified methadone prescription does not exclude later non-CHI-identified prescriptions. Hence, once included in the cohort, clients have remained in follow up.

Confounding between DRD-risk and the client's daily dose quintile cannot be ruled out.^18^ Age >45 years and prescribed daily dose >90 mg may be markers for harder-to-support clients whose opioid dependency is chronic, who have physical comorbidities, notably circulatory and digestive system diseases, or coprescriptions for mental or physical ill health.^8^

Finally, we did not request that methadone clients' coprescriptions^34^ for benzodiazepines, antiviral medications, mirtazapine, amitriptyline, sertraline or macrolide antibiotics (to name but a few) be linked in because the added time and complexity would not have been warranted given that illicit supplies would have remained unaccounted for: and constitute most of the benzodiazepines present at Scotland's DRDs.^8^

## 5 Conclusions

Scotland's 2009–2015 methadone-prescription cohort helps to explain why UK official statistics on DRDs and opioid-specific deaths in the second decade of 21^st^ show stark increases by age-group, and disproportionately so for females.^10^ Methadone-prescription clients, including CHI unidentified, during July 2009 to June 2015 accounted for around 70% (546/0.80 coverage] of Scotland's 983 methadonespecific DRDs in July 2009 to 31 December 2015.

Our analyses shed an uncompromising light on the wave of age-related, opioid-specific DRDs that overwhelms survivors from the UK's heroin injector epidemics of the early 1980s. Clinicians have a difficult balance to strike. Unlike record-linkage studies, official statistics do not chart clients' non-DRDs, which predominate over DRDs by at least 3:1 as clients age beyond 45 years. Sustained methadone maintenance, as recommended,^27^ has halved the DRD rate that clients would otherwise have experienced at an age when DRDs did predominate.

Urgently, interventions are needed to de-escalate ageing methadone clients' risk not only of methadone-specific DRD but also of their major causes of non-DRDs.^19^ Practitioners must balance: moderation of clients' daily dose of methadone, ideally to below 90 mg if clients are willing; review of circulatory or digestive comorbidities that, respectively, risk sudden death masquerading as methadone-specific DRD or prolongation of methadone's half-life; support for smoking cessation to manage better the client's respiratory and circulatory diseases; hepatitis C virus clearance by directly acting antiviral therapy; and review of medications prescribed for psychiatric and physical comorbidities for possible interactions with methadone.^34^

Methadone exhibits large interindividual variation in response, a narrow therapeutic index and interacts with a range of other drugs commonly prescribed for ageing methadone-prescription clients' comorbidities. Too little is known about the age-related or sexed pharmacogenomics, pharmacokinetics and pharmacodynamics of methadone.^22,32,41^

## Supplementary Material

Supplementary materials

## Figures and Tables

**Table 1:** Descriptive statistics for Scotland's 2009–2015 Community Health Index (CHI)-identified methadone-prescription cohort

Descriptor	2009-2015 CHI-identified methadone-prescription cohort	
	All clients	With actual or recovered daily dose at first CHI-identified prescription
Clients	36 347	26 533
Person-years	192 928	144 697
Deaths	3180	2342
DRDs (%)	1323 (42%)	995 (42%)
Methadone-specific DRDs (% of all DRDs)	546 (41%)	420 (42%)
Heroin-specific DRDs (% of all DRDs)	320 (24%)	241 (24%)
Heroin + methadone DRDs (% of all DRDs)	309 (23%)	219 (22%)
**Sex**		
Females (%)	12 096 (33%)	8744 (33%)
Males (%)	24 251 (67%)	17 789 (67%)
**Prescription source**		
GP-prescriber	23 604 (65%)	19 184 (72%)
Other-source prescriber	12 743 (35%)	7349 (28%)
**Mean (standard deviation)**		
Age at accrual	34.8 y (7.9)	34.9 y (7.8)
1^st^ actual or recovered daily dose at accrual	61.7 mg (34.2) 11 055 clients with actual daily dose	62.8 mg (33.2) 26 533 clients with actual or recovered daily dose
Quantity methadone at 1^st^ CHI-identified script in accrual month	1181 mg (1116)	1260 mg (1112)
**Client's number of CHI-identified prescriptions with actual or recovered daily dose in accrual month**		
1		17 349 (65%)
2		6393 (24%)
3		1926 (7%)
4		601 (2%)
5 or more		264 (1%)
**Clients' accrual era**		
July–December 2009	20 728	16 350
2010	6489	4428
2011	2993	1973
2012	1938	1274
January–June 2013	813	517
July–December 2013	985	577
2014	1661	979
January–June 2015	740	435

DRD, drug-related death.

**Table 2:** Death rates (95% CI) for 1000 person-years for all 36 347 clients in Scotland's 2009-2015 Community Health Index (CHI)-identified methadone-prescription cohort

All CHI-identified methadone-prescription clients								
Covariate	Clients (%)	Total CHI-identified prescriptions	Person-years	Non-DRDs	DRDs	Methadone-specific DRDs [M] (%)	Heroin-specific DRDs [H]	M + H DRDs
Scotland wide	36 347	1 931 062	192 928	1857	1323	546	320	309
Death rates (95% CI)				9.6 (9.2–10.1)	6.9 (6.5–7.2)	2.8 (2.6–3.l)	1.7 (1.5–1.9)	1.6 (1.4–1.8)
***Prescription source***								
GP	23 604 (65%)	1419 112	130 856	1351 (73%)	846 (64%)	347 (64%)	226	165
Death rates (95% CI)				10.3(9.8–10.9)	6.5 (6.0–6.9)	2.7 (2.4-2.9)		
Other	12 743 (35%)	511 950	62 072	506 (27%)	477 (36%)	199 (36%)	94	144
Death rates (95% CI)				8.2 (7.5–8.9)	7.7 (7.0–8.4)	3.2 (2.8–3.7)		
***Sex***								
Male	24 251 (67%)	1 253 996	128 042	1315 (71%)	908 (69%)	339 (62%)	252	218
Death rates (95% CI)				10.3 (9.7–10.8)	7.1 (6.6–7.6)	2.6 (2.4–2.9)		
Female	12 096 (33%)	677 066	64 886	542 (29%)	415 (31%)	207 (38%)	68	91
Death rates (95% CI)				8.4 (7.7–9.1)	6.4 (5.8–7.0)	3.2 (2.8–3.7)		
***Age group at baseline (at accrual)***								
15–24y	2965 (8%)	131 287	14 941	50 (3%)	64 (5%)	20 (4%)	23	15
Death rates (95% CI)				3.3 (2.5–4.4)	4.3 (3.4–5.5)	1.3 (0.9–2.1)		
25–34y	15 957 (44%)	862 064	86 825	408 (21%)	504 (38%)	163 (30%)	154	137
Death rates (95% CI)				4.7 (4.3–5.2)	5.8 (5.3–6.3)	1.9 (1.6–2.2)		
35–44y	13 416 (37%)	751 673	72 207	803 (42%)	567 (43%)	264 (48%)	116	118
Death rates (95% CI)				11.1 (10.4–11.9)	7.9 (7.2–8.5)	3.7 (3.2–4.1)		
45+ years	4009 (11%)	186 038	18 956	596 (34%)	188 (14%)	99 (18%)	27	39
Death rates (95% CI)				31.4 (29.0–34.1)	9.9 (8.6–11.4)	5.2 (4.3–6.4)		
***Quintiles for baseline quantity of prescribed methadone defined by 1^st^ CHI-identified prescription*** ^1^								
qQ1: 5–270 mg	7282 (20%)	321 751	32 396	368 (20%)	224 (17%)	77 (14%)	75	41
Death rates (95% CI)				11.4 (10.3–12.6)	6.9 (6.1–7.9)	2.4 (1.9–3.0)		
qQ2: 271–645 mg	7259 (20%)	378 255	37 117	384 (21%)	271 (20%)	92 (17%)	77	68
Death rates (95% CI)				10.3 (9.4–11.4)	7.3 (6.5–8.2)	2.5 (2.0–3.0)		
qQ3: 646–1120 mg	7298 (20%)	438 174	40 542	351 (19%)	250 (19%)	85 (16%)	71	66
Death rates (95% CI)				8.7 (7.8–9.6)	6.2 (5.4–7.0)	2.1 (1.7–2.6)		
qQ4: 1121–1960 mg	7915 (22%)	450 504	44 478	413 (22%)	288 (22%)	128 (23%)	62	69
Death rates (95% CI)				9.3 (8.4–10.2)	6.5 (5.8–7.3)	2.9 (2.4–3.4)		
qQ5: >1960 mg	6593 (18%)	351 378	38 395	341 (18%)	290 (22%)	164 (30%)	35	65
Death rates (95% CI)				8.9 (8.0–9.9)	7.6 (6.7–8.5)	4.3 (3.7–5.0)		

1 Quintiles for baseline quantity of prescribed methadone defined by 1^st^ CHI-identified prescription, are as follows qQ1: 5–270 mg; qQ2: 271–645 mg; qQ3: 646–1120 mg; qQ4: 1121–1960 mg; qQ5: >1960 mg.

CI, confidence interval; DRD, drug-related death.

**Table 3:** Death rates (95% CI) for 1000 person-years for 26 533 clients in Scotland's 2009-2015 Community Health Index (CHI)-identified methadone-prescription cohort with actual or recovered daily dose at first CHI-identified prescription

Clients with actual or recovered daily dose at first CHI-identified prescription								
Covariate	Clients (%)	Total CHI-identified prescriptions	Person-years	Non-DRDs (%)	DRDs (%)	Methadone-specific DRD [M] (%)	Heroin-specific DRD [H]	M + H DRDs
Scotland wide	26 533	1 510 417	144 697	1347	995	420	241	219
Death rates (95% CI)				9.3 (8.8–9.8)	6.9 (6.5–7.3)	2.9 (2.6–3.2)		
***Prescription source***								
GP	19 184 (72%)	1 178 699	107 248	1030 (76%)	706 (71%)	293 (70%)	184	136
Death rates (95% CI)				9.6 (9.0–10.2)	6.6 (6.1–7.1)	2.7 (2.4–3.1)		
Other	7349 (28%)	331 718	37 449	317 (24%)	289 (29%)	127 (30%)	57	83
Death rates (95% CI)				8.5 (7.6–9.5)	7.7 (6.9–8.7)	3.4 (2.9–4.0)		
***Sex***								
Male	17 789 (67%)	985 116	96 377	970 (72%)	696 (70%)	269 (64%)	196	156
Death rates (95% CI)				10.1 (9.5–10.7)	7.2 (6.7–7.8)	2.8 (2.5–3.2)		
Female	8744 (33%)	525 301	48 320	377 (28%)	299 (30%)	151 (36%)	45	63
Death rates (95% CI)				7.8 (7.0–8.6)	6.2 (5.5–6.9)	3.1 (2.7–3.7)		
***Age group at baseline (at accrual)***								
15-24y	1991 (8%)	96 353	10 355	36 (3%)	40 (4%)	13 (3%)	14	8
Death rates (95% CI)				3.5 (2.5–4.8)	3.9 (2.8–5.3)	1.3 (0.7–2.2)		
25–34y	11 567 (44%)	666 980	64 492	299 (22%)	370 (37%)	123 (29%)	115	93
Death rates (95% CI)				4.6 (4.1–5.2)	5.7 (5.2–6.4)	1.9 (1.6–2.3)		
35–44y	10 054 (38%)	599 478	55 553	594 (44%)	435 (44%)	206 (49%)	91	88
Death rates (95% CI)				10.7 (9.9–11.6)	7.8 (7.1–8.6)	3.7 (3.2–4.3)		
45+y	2921 (11%)	147 606	14 297	418 (31%)	150 (15%)	78 (19%)	21	30
Death rates (95% CI)				29.2 (26.6–32.2)	10.4 (8.9–12.3)	5.4 (4.4–6.8)		
***Quintiles (qQ) for baseline quantity of prescribed methadone defined by 1^st^ CHI-identified prescription*** ^1^								
qQ1: 5–336 mg	5318 (20%)	251 575	25 025	274 (20%)	179 (18%)	57 (14%)	64	29
Death rates (95% CI)				11.0 (9.9–12.3)	7.2 (6.2–8.3)	2.3 (1.8–3.0)		
qQ2: 337–765 mg	5296 (20%)	308 169	28 345	268 (20%)	207 (21%)	66 (16%)	65	53
Death rates (95% CI)				9.5 (8.4–10.7)	7.3 (6.4–8.4)	2.3 (1.8–3.0)		
qQ3: 766–1260 mg	5 714 (22%)	368 734	32 505	291 (22%)	191 (19%)	82 (20%)	48	38
Death rates (95% CI)				9.0 (8.0–10.0)	5.9 (5.1–6.8)	2.5 (2.0–3.1)		
qQ4: 1261–1960 mg	4979 (19%)	290 517	28 258	250 (19%)	182 (18%)	80 (19%)	35	50
Death rates (95% CI)				8.9 (7.8–10.0)	6.4 (5.6–7.4)	2.8 (2.3–3.5)		
Q5: >1960 mg	5226 (20%)	291422	30 564	264 (20%)	236 (24%)	135 (32%)	29	49
Death rates (95% CI)				8.6 (7.7–9.7)	7.7 (6.8–8.8)	4.4 (3.7–5.2)		
***Quintiles (dQ) for baseline daily dose of prescribed methadone defined by 1^st^ CHI-identified prescription*** ^2^								
dQ1: (1–34.5) mg	5307 (20%)	244 273	26 499	327 (24%)	152 (15%)	41 (10%)	54	29
Death rates (95% CI)				12.3 (11.1–13.8)	5.7 (4.9–6.7)	1.6 (1.1–2.1)		
dQ2: (34.5–50) mg	6287 (24%)	336 090	32 918	294 (22%)	212 (22%)	72 (17%)	62	45
Death rates (95% CI)				8.3 (7.4–9.4)	6.3 (5.5–7.3)	2.2 (1.7–2.8)		
dQ3: (50–70) mg	5892 (22%)	348 984	32 859	274 (20%)	208 (21%)	87 (21%)	52	55
Death rates (95% CI)				8.3 (7.4–9.4)	6.3 (5.5–7.3)	2.7 (2.2–3.3)		
dQ4: (70–90) mg	4645 (18%)	288 961	26 658	234 (17%)	199 (20%)	91 (22%)	44	46
Death rates (95% CI)				8.8 (7.7–10.0)	7.5 (6.5–8.6)	3.4 (2.8–4.2)		
dQ5: >90 mg	4402 (17%)	292 109	25 763	218 (16%)	224 (23%)	129 (31%)	29	44
Death rates (95% CI)				8.5 (7.4–9.7)	8.7 (7.6–9.9)	5.0 (4.2–6.0)		

1 Quintiles (qQ) for baseline quantity of prescribed methadone defined by 1^st^ CHI-identified prescription are as follows qQ1: 5–336 mg; qQ2: 337-765 mg; qQ3: 766–1260 mg; qQ4: 1261–1960 mg; Q5: >1960 mg.

2 Quintiles (dQ) for baseline daily dose of prescribed methadone defined by 1^st^ CHI-identified prescription are as follows dQ1: [1-34.5] mg; dQ2: (34.5-50] mg; dQ3: (50-70] mg; dQ4: (70-90] mg; dQ5: >90 mg. Cl, confidence interval; DRD, drug-related death.

**Table 4:** Proportional hazards regression for methadone-specific DRDs for 36 347 clients with 192 928 person-years of follow up in Scotland's 2009–2015 Community Health Index (CHI)-identified methadone-prescription cohort, incorporating quintiles for quantity of methadone prescribed at first CHI-identified methadone-prescription (qQ)

Quintiles for baseline quantity of prescribed methadone at qQ (qQ regression χ^2^ of 33.40 on 4 degrees of freedom; P < .00001)		
Events	546 methadone-specific DRDs	
Covariates	HR	95% confidence interval for HR; P-value vs baseline
Prescription source (baseline: GP prescriber)		
Other	1.36	1.14–1.62; P = .001
Sex (baseline: male)		
Female	1.37	1.15–1.63; P < .001
Age group at accrual (baseline: 25-34 y)		
<25 y	0.67	0.42–1.07
25–34 y	1.00	Baseline
35–44 y	2.02	1.66–2.46; P < .001
45+ y	2.94	2.29–3.78; P < .001
Quintiles for prescribed quantity at accrual (qQ1 as baseline)		
qQ1: 5–270 mg	1.00	Baseline
qQ2: 271–645 mg	0.98	0.72–1.33
qQ3 646–1120 mg	0.79	0.58–1.07
qQ4: 1121–1960 mg	1.08	0.81–1.44
qQ5: >1960 mg	1.61	1.22–2.11; P = .001

CI, confidence interval; DRD, drug-related death; HR, hazard ratio.

**Table 5:** Proportional hazards regression for methadone-specific DRDs for 26 533 clients Scotland's 2009**–**2015 Community Health Index (CHI)-identified methadone-prescription cohort with actual or recovered daily dose of methadone at first CHI-identified methadone prescription and 144 697 person-years of follow up

Quintiles for actual or recovered daily dose of methadone at 1^st^ CHI-identified prescription (dQ) dQ regression χ^2^ of 58.99 on 4 degrees of freedom; P < .00001		
Events	420 methadone-specific DRDs	
Covariates	HR	95% CI for HR P-value vs baseline
Prescription source (baseline: GP prescriber)		
Other	1.32	1.07–1.63; P = .009
Sex (baseline: male)		
Female	1.29	1.05–1.58; P = .013
Age group at accrual (baseline: 25-34 y)		
<25 y	0.67	0.38–1.19
25–34 y	1.00	Baseline
35–44 y	2.00	1.60–2.50; P < .001
45+ y	3.15	2.37–4.19; P < .001
Quintiles for prescribed quantity at accrual (qQ1 as baseline)		
dQ1: [1–34.5] mg	1.00	Baseline
dQ2: (34.5–50] mg	1.39	0.95–2.04
dQ3 (50–70] mg	1.68	1.15–2.43; P = .007
dQ4: (70–90] mg	2.16	1.49–3.13; P < .001
dQ5: >90 mg	3.15	2.21–4.48; P < .001

CI, confidence interval; DRD, drug-related death; HR, hazard ratio.

## Data Availability

Data were made available for analysis after our application to Scotland's Electronic Data Research and Innovation Service (eDRIS) was approved by Scotland's Public Benefit and Privacy Panel (PBPP). The data can be accessed by other research teams who submit a successful PBPP application via eDRIS but are not available directly from the authors.

## What is already known about this subject

- Opioid substitution therapy halves clients' drug-related death rate.
- However, age-related increases drug-related and methadonespecific deaths persist.
- Opioid cohorts lack substantial data on prescribed daily dose of methadone.

## What this study adds

- Validated simple rules for recovery of prescribed daily dose of methadone.
- Baseline daily dose of prescribed methadone available for 73% of Scotland's methadone-client cohort (2009–2015).
- Top quintile for prescribed daily dose (>90 mg per day) associated with increased hazard for methadone-specific death vs 50–70 mg; 3 times greater risk of methadonespecific death at 45+ years vs 25–34 years; females may be at greater risk of methadone-specific death.
