# Supplementary material for: Scotland's 2009–2015 methadone-prescription cohort: Quintiles for daily dose of prescribed methadone and risk of methadone-specific death
Source: Br J Clin Pharmacol. Author manuscript; Available in PMC 2022 Jan 5. (PMC7612180; doi:10.1111/bcp.14432)
Supplement: Supplementary materials [file EMS140626-supplement-Supplementary_Materials.pdf]

39. Hill AB. The environment and disease: association or causation? *Proc R Soc Med*. 1965;58(5):295-300.
40. Public Health England. *Non-medical prescribing in the management of substance misuse*. London: Public Health England; July 2014. See https://www.gov.uk/government/publications/non-medical-prescribing-in-the-management-of-substance-misuse
41. Lugo RA, Satterfield KL, Kern SE. Pharmacokinetics of methadone. *J Pain Palliat Care Pharmacother*. 2005;19(4):13-24.

How to cite this article: Gao L, Robertson JR, Bird SM. Scotland's 2009–2015 methadone-prescription cohort: Quintiles for daily dose of prescribed methadone and risk of methadone-specific death. *Br J Clin Pharmacol*. 2021;87: 652–673. https://doi.org/10.1111/bcp.14432

APPENDIX 1: Rules for recovery of daily dose

Accepted rules for recovery of daily dose: The performance of recovery rules, see Table A1, was tested in alternative settings:

i. Scotland's 2013–15 derivation subcohort, as defined in **Methods**: all Community Health Index (CHI)-identified prescriptions with daily dose in clients' accrual month.
ii. Scotland's 2009–2015 methadone-prescription client cohort: all CHI-identified prescriptions with daily dose in clients' cohort-entry month.

As shown in Table A1, recovery rules were generally of the form "quantity divided by D(i)" where the value, D (i), depended on the number (i) of instalments, with extra conditions needed only when the number of instalments was 4 or 7.

To be accepted, a recovery rule for i instalments had to match 75% of actual daily doses issued in i instalments in the accrual month for clients in the derivation subcohort. The accepted rules recovered 90% of 7,964 relevant daily doses for clients in the derivation subcohort; and 88% of 8389 relevant daily doses in the cohort entry month for Scotland's 2009–2015 methadone-prescription client cohort. Recovery rate dropped below 75% in Scotland's 2009–2015 cohort only twice: for 4 instalments (71%) and 35 instalments (67%).

TABLE A1 Performance of accepted recovery rules for daily dose

Number of instalments with acceptable recovery rule	Accepted recovery rule for daily dose	Performance of per-instalment recovery rule	Scotland's 2009–2015 methadone-prescription cohort: Accrual month	2013–2015 derivation subcohort: Accrual month
4	If number of instalments is the same for every prescription in client's accrual month: divide quantity by 28. Otherwise divide quantity by 4 unless answer exceeds 200 mg, in which case divide by 28	% correct *vs* actual daily dose	71% (1251/1765)	76% (1374/1805)
		Increase in daily dose by applying rule	Increased from 1765 to 5276	Increased from 1805 to 4413
		Number other-source prescriptions with daily dose by applying rule	1305	1498
7	Divide quantity by 7 unless answer exceeds 200 mg, in which case divide by 49, i.e. 7 × 7.	% correct *vs* actual daily dose	90% (1818/2020)	92% (1289/1402)
		Increase in daily dose by applying rule	Increased from 2020 to 7550	Increased from 1402 to 4346
		Number other-source prescriptions with daily dose by applying rule	1274	828
14	Divide quantity by 14	% correct *vs* actual daily dose	94% (1950/2081)	95% (1710/1802)
		Increase in daily dose by applying rule	Increased from 2081 to 9416	Increased from 1802 to 8229
		Number other-source prescriptions with daily dose by applying rule	3979	3921
21	Divide quantity by 21	% correct *vs* actual daily dose	86% (331/ 386)	90% (353/ 391)
		Increase in daily dose by applying rule	Increased from 386 to 1231	Increased from 391 to 1072
		Number other-source prescriptions with daily dose by applying rule	135	132

TABLE A1 (Continued)

Number of instalments with acceptable recovery rule	Accepted recovery rule for daily dose	Performance of per-instalment recovery rule	Scotland's 2009–2015 methadone-prescription cohort: Accrual month	2013–2015 derivation subcohort: Accrual month
28	Divide quantity by 28	% correct *vs* actual daily dose	96% (1863/1950)	97% (2289/2365)
		Increase in daily dose by applying rule	Increased from 1950 to 6461	Increased from 2365 to 6924
		Number other-source prescriptions with daily dose by applying rule	2730	2893
35	Divide quantity by 35	% correct *vs* actual daily dose	67% (24/ 36)	78% (21/ 27)
		Increase in daily dose by applying rule	Increased from 36 to 65	Increased from 27 to 53
		Number other-source prescriptions with daily dose by applying rule	5	0
42	Divide quantity by 42	% correct *vs* actual daily dose	78% (59/ 76)	87% (72/ 83)
		Increase in daily dose by applying rule	Increased from 76 to 118	Increased from 83 to 91
		Number other-source prescriptions with daily dose by applying rule	5	0
56	Divide quantity by 56	% correct *vs* actual daily dose	96% (72/ 75)	98% (87/ 89)
		Increase in daily dose by applying rule	Increased from 75 to 112	Increased from 89 to 109
		Number other-source prescriptions with daily dose by applying rule	11	7
By applying the above 8 accepted rules for recovery of daily dose when number of instalments is: 4, 7, 14, 21, 28, 35, 42 or 56		% correct *vs* actual daily dose	88% (7368/8389)	90% (7195/7964)
		Increase in daily dose by applying rule	Increased from 8389 to 30 229	Increased from 7964 to 25 237
		Number other-source prescriptions with daily dose by applying rule	9444	9279

APPENDIX 2: Proportional hazards (PH) analysis for all DRDs

Baseline quintile for prescribed quantity: For all DRDs, Table A2 shows that the interaction between sex and age group at accrual is highly statistically significant (*P* = .0026) and signals that female clients' reduced DRD hazard (overall HR, 0.82) is reversed for older clients. Male clients' DRD risk increases with age, being significantly greater for males aged 45+ years at accrual (HR, 1.59) than if aged 25–34 years; and greater also than for males aged 35–44 years (HR = 1.59/1.25 or 1.27, 95% CI: 0.99 to 1.63). There is some indication that clients in qQ3 or qQ4 at accrual have reduced DRD hazard (HR = 0.8) compared to qQ1. Clients whose prescription source was non-GP had significantly higher DRD risk (HR, 1.31).

For methadone-specific DRDs, interaction between sex and age group is unnecessary; females have higher HR (1.4) than males; HRs increase very steeply with age group at accrual, being 3-fold higher for clients 45+ years at accrual and 2-fold greater for clients aged 35–44 years than if 25–34 years at accrual. Only the top quintile for quantity of prescribed methadone was associated with a significantly increased HR compared to qQ3.

Baseline quintile for recovered or actual daily dose: For all DRDs, the significant sex by age group interaction in Table A3 confirms that females enjoy an importantly reduced DRD risk younger than 35 years compared to males but, thereafter, the female advantage is neutralized. Males' DRD risk increases significantly at 35+ years. The HR associated with dQ5, top quintile for daily dose at first CHI-identified prescription, was significantly greater than for dQ3 (HR = 1.43/1.06 or 1.35; 95% CI: 1.00 to 1.81). Higher DRD risk was associated with clients whose prescriber was other source.

For methadone-specific DRDs, females are disadvantaged (HR 1.3, 95% CI: 1.0–1.6) and the steepness of increase in HRs, both age related and by quintile for 1st daily dose, is much greater than for all DRDs. By age group, HR was 3-fold higher (95% CI: 2.4 to 4.2) at age 45+ years than at 25–34 years. For dQ5, HR was also 3-fold higher (95% CI: 2.2 to 4.5) than for dQ1 and significantly greater than for dQ3 (HR = 3.15/1.68 or 1.88; 95% CI: 1.13 to 3.12), itself significantly greater than dQ1. Finally, higher HR for methadone-specific DRD was associated with other-source prescribers.

TABLE A2 Proportional hazards regressions for Scotland's 2009–2015 Community Health Index (CHI)-identified methadone-prescription cohort of 36 347 clients and 192 928 py, incorporating quintiles for quantity of methadone prescribed at first CHI-identified methadone-prescription

Quintiles for baseline quantity of prescribed methadone at 1st CHI-identified prescription				
**Events**	**1323 DRDs**		**546 methadone-specific DRDs**	
**Covariates**	HR	95% CI for HR; *P*-value *vs* baseline	HR	95% CI for HR; *P*-value *vs* baseline
qQ regression, 4df	***χ^2 11.87; P = .018***		***χ^2 33.40; P < .00001***	
**Prescription source (baseline: GP prescriber)**				
Other	1.31	1.17–1.47; *P* < .001	1.36	1.14–1.62; *P* = .001
**Sex (baseline: male)**				
Female	0.95	0.85–1.07	1.37	1.15–1.63; *P* < .001
**Age group at accrual (baseline: 25–34 y)**				
<25 y	0.72	0.56–0.94; *P* = .015	0.67	0.42–1.07
25–34 y	1.00		1.00	
35–44 y	1.38	1.23–1.56; *P* < .001	2.02	1.66–2.46; *P* < .001
45+ y	1.79	1.51–2.12; *P* < .001	2.94	2.29–3.78; *P* < .001
**Quintiles for prescribed quantity at accrual (qQ1 as baseline)**				
qQ1: 5–270 mg	1.00		1.00	
qQ2: 271–645 mg	0.99	0.83–1.19	0.98	0.72–1.33
qQ3 646–1120 mg	0.80	0.67–0.96; *P* = 0.015	0.79	0.58–1.07
qQ4: 1121–1960 mg	0.83	0.70–0.99; *P* = 0.040	1.08	0.81–1.44
qQ5: >1960 mg	0.97	0.81–1.15	1.61	1.22–2.11; *P* = .001
Now including interaction for sex by age group at accrual on 3 degrees of freedom (df)				
Interaction regression χ^2 on 3df	***14.27; P = .0026***		***4.00; P = .26***	
**Prescription source (baseline: GP prescriber)**				
Other	1.31	1.17–1.47; *P* < .001	1.36	1.14–1.62; *P* = .001
**Sex (baseline: male)**				
Female	0.82	0.68–0.99; *P* = .035	1.21	0.89–1.66

TABLE A2 (Continued)

Quintiles for baseline quantity of prescribed methadone at 1st CHI-identified prescription				
**Events**	**1323 DRDs**		**546 methadone-specific DRDs**	
**Age group at accrual (baseline: 25–34 y)**				
<25 y	0.92	0.66–1.28	0.84	0.44–1.62
25–34 y	1.00		1.00	
35–44 y	1.25	1.08–1.45; *P* = .002	1.81	1.40–2.32; *P* < .001
45+ y	1.59	1.30–1.95; *P* < .001	2.93	2.15–3.99; *P* < .001
**Interaction between female & age group at accrual (baseline: female & 25–34 y)**				
<25 y	0.61	0.36–1.05; *P* = .074	0.69	0.27–1.76
25–34 y	1.00		1.00	
35–44 y	1.37	1.06–1.78; *P* = .016	1.34	0.90–2.00
45+ y	1.46	1.02–2.10; *P* = .040	0.98	0.58–1.66
**Quintiles for prescribed quantity at accrual (qQ1 as baseline)**				
qQ1: 5–279 mg	1.00		1.00	
qQ2: 271–645 mg	0.99	0.83–1.19	0.98	0.72–1.33
qQ3: 646–1120 mg	0.80	0.67–0.96; *P* = .015	0.78	0.57–1.07
qQ4: 1121–1960 mg	0.83	0.70–0.99; *P* = .041	1.08	0.81–1.48; *P* = .001
qQ5: >1960 mg	0.97	0.82–1.16	1.60	1.22–2.11; *P* = .001

CI, confidence interval; DRD, drug-related death; HR, hazard ratio

TABLE A3 Proportional hazards regressions for Scotland's restricted 2009–2015 Community Health Index (CHI)-identified methadone-prescription cohort of 26 533 clients and 144 697 py, with actual or recovered daily dose of methadone prescribed at first CHI-identified methadone prescription

Quintiles for actual or recovered daily dose of prescribed methadone at 1st CHI-identified prescription				
**Events**	**995 DRDs**		**420 methadone-specific DRDs**	
**Covariates**	HR	95% CI for HR; *P*-value *vs* baseline	HR	95% CI for HR; *P*-value *vs* baseline
**Prescription source (baseline: GP prescriber)**				
Other	1.25	1.09–1.44; *P* = .001	1.32	1.07–1.63; *P* = .009
**Sex (baseline: male)**				
Female	0.92	0.80–1.05	1.29	1.05–1.58; *P* = 0.013
**Age group at accrual (baseline: 25–34 y)**				
<25 y	0.70	0.50–0.97; *P* = .033	0.67	0.38–1.19
25–34 y	1.00		1.00	
35–44 y	1.37	1.19–1.58; *P* < .001	2.00	1.60–2.50; *P* < .001
45+ y	1.91	1.58–2.31; *P* < .001	3.15	2.37–4.19; *P* < .001
**Quintiles for prescribed daily dose at accrual (qQ1 as baseline)**				
dQ1: [1–34.5] mg	1.00		1.00	
dQ2: (34.5–50] mg	1.10	0.89–1.35	1.39	0.95–2.04
dQ3 (50–70] mg	1.06	0.86–1.31	1.68	1.15–2.43; *P* = .007
dQ4: (70–90] mg	1.24	1.00–1.53; *P* = .046	2.16	1.49–3.13; *P* < .001
dQ5: >90 mg	1.43	1.17–1.76; *P* = .001	3.15	2.21–4.48; *P* < .001
Now including interaction for sex by age group at accrual on 3 degrees of freedom (df)				
Interaction regression χ^2 on 3df	***11.13; P = .011***		***6.52; P = .09***	

(Continues)

TABLE A3 (Continued)

Quintiles for actual or recovered daily dose of prescribed methadone at 1st CHI-identified prescription				
**Events**	**995 DRDs**		**420 methadone-specific DRDs**	
**Prescription source (baseline: GP prescriber)**				
Other	1.26	1.09–1.44; *P* = .001	1.33	1.08–1.64; *P* = .008
**Sex (baseline: male)**				
Female	0.78	0.63–0.98; *P* = .031	1.20	0.83–1.72
**Age group at accrual (baseline: 25–34 y)**				
<25 y	0.92	0.62–1.38	1.02	0.49–2.13
25–34 y	1.00		1.00	
35–44 y	1.23	1.04–1.46; *P* = .014	1.80	1.35–2.40; *P* < .001
45+ y	1.76	1.40–2.20; *P* < .001	3.34	2.37–4.70; *P* < .001
**Interaction between female & age group at accrual (baseline: Female & 25–34 y)**				
<25 y	0.55	0.28–1.10	0.43	0.13–1.39
25–34 y	1.00		1.00	
35–44 y	1.42	1.05–1.92; *P* = .023	1.33	0.84–2.10
45+ y	1.32	0.86–2.02	0.78	0.42–1.47
**Quintiles for prescribed daily dose at accrual (qQ1 as baseline)**				
dQ1: [1–34.5] mg	1.00		1.00	
dQ2: (34.5–50] mg	1.10	0.89–1.35	1.39	0.95–2.05
dQ3 (50–70] mg	1.06	0.86–1.31	1.67	1.15–2.43; *P* = .007
dQ4: (70–90] mg	1.24	1.00–1.53; *P* = .048	2.15	1.49–3.12; *P* < .001
dQ5: >90 mg	1.43	1.16–1.76; *P* < .001	3.15	2.21–4.47; *P* < .001

CI, confidence interval; DRD, drug-related death; HR, hazard ratio

APPENDIX 3: Sensitivity analyses

TABLE A4 Proportional hazards regressions for Scotland's restricted 2009–2015 Community Health Index (CHI)-identified methadone-prescription cohort of 26 533 clients and 144 697 py, with actual or recovered daily dose of methadone prescribed at first CHI-identified methadone prescription and current age group

Quintiles for daily dose of prescribed methadone at 1st CHI-identified prescription				
Events	**995 DRDs**		**420 methadone-specific DRDs**	
**Covariates**	HR	95% CI for HR; *P*-value *vs* baseline	HR	95% CI for HR; *P*-value *vs* baseline
dQ regression, 4df	***χ^2 16.34; P = .0026***		***χ^2 58.59; P < .00001***	
**Prescription source (baseline: GP prescriber)**				
Other	1.27	1.10–1.45; *P* = .001	1.34	1.08–1.65; *P* = .007
**Sex (baseline: male)**				
Female	0.92	0.80–1.06	1.28	1.05–1.56; *P* = .016
**Current age group (baseline: 25–34 y)**				
<25 y	0.71	0.43–1.16	0.58	0.23–1.43
25–34 y	1.00		1.00	
35–44 y	1.36	1.16–1.58; *P* < .001	1.70	1.32–2.18; *P* < .001
45+ y	2.10	1.77–2.50; *P* < .001	3.15	2.40–4.13; *P* < .001
**Quintiles for prescribed daily dose at accrual (dQ1 as baseline)**				
dQ1: [1–35] Mg	1.00		1.00	
dQ2: (35–50] mg	1.09	0.89–1.34	1.42	0.98–2.06

TABLE A4 (Continued)

Quintiles for daily dose of prescribed methadone at 1st CHI-identified prescription				
Events	**995 DRDs**		**420 methadone-specific DRDs**	
dQ3 (50–70] mg	1.06	0.86–1.29	1.65	1.16–2.35; *P* = .006
dQ4: (70–90] mg	1.24	1.01–1.51; *P* = .043	2.11	1.49–3.01; *P* < .001
dQ5: >90 mg	1.43	1.17–1.75; *P* < .001	3.10	2.22–4.33; *P* < .001
Now including interaction for sex by current age group on 3 degrees of freedom (df)				
Interaction regression, 3df	***χ^2 10.18; P = .017***		***χ^2 6.49; P = .090***	
**Prescription source (baseline: GP prescriber)**				
Other	1.27	1.10–1.46; *P* = .001	1.34	1.08–1.65; *P* = .007
**Sex (baseline: male)**				
Female	0.70	0.54–0.91; *P* = .007	1.06	0.70–1.60
**Current age group (baseline: 25–34 y)**				
<25 y	0.93	0.51–1.72	1.16	0.42–3.20
25–34 y	1.00		1.00	
35–44 y	1.18	0.99–1.42	1.46	1.06–2.01; *P* = .020
45+ y	1.84	1.50–2.26; *P* < .001	2.97	2.12–4.16; *P* < .001
**Interaction between female & current age group (baseline: female & 25–34 y)**				
<25 y	0.58	0.21–1.61	0.18	0.02–1.65
25–34 y	1.00		1.00	
35–44 y	1.52	1.09–2.11; *P* = .013	1.46	0.88–2.43
45+ y	1.52	1.05–2.22; *P* = .028	1.12	0.64–1.97
**Quintiles for prescribed daily dose at accrual (dQ1 as baseline)**				
dQ1: [1–34.5] mg	1.00		1.00	
dQ2: (34.5–50] mg	1.09	0.89–1.34	1.42	0.97–2.06
dQ3 (50–70] mg	1.06	0.86–1.29	1.65	1.15–2.35; *P* = .006
dQ4: (70–90] mg	1.23	1.01–1.51; *P* = .045	2.11	1.48–3.00; *P* < .001
dQ5: >90 mg	1.43	1.17–1.74; *P* < .001	3.10	2.22–4.33; *P* < .001

CI, confidence interval; DRD, drug-related death; HR, hazard ratio

TABLE A5 Proportional hazards regressions for Scotland's restricted 2009–2015 Community Health Index (CHI)-identified methadone-prescription cohort of 19 184 GP clients and 107 284 py, with actual or recovered daily dose of methadone prescribed at first CHI-identified methadone prescription and current age group

**Quintiles for daily dose of prescribed methadone at 1st CHI-identified prescription for GP clients**				
Events	**706 DRDs**		**293 methadone-specific DRDs**	
**Covariates**	HR	95% CI for HR; *P*-value *vs* baseline	HR	95% CI for HR; *P*-value *vs* baseline
dQ regression, 4df	***χ^2 14.45; P = .0060***		***χ^2 44.60; P < .00001***	
**Sex (baseline: Male)**				
Female	0.91	0.78–1.08	1.38	1.09–1.76; *P* = .008
**Current age group (baseline: 25–34 y)**				
<25 y	.79	.43–1.46	.58	.18–1.86
25–34 y	1.00		1.00	
35–44 y	1.30	1.08–1.57; *P* = .005	1.49	1.10–2.00; *P* = .009
45+ y	1.91	1.55–2.34; *P* < .001	2.57	1.86–3.54; *P* < .001
**Quintiles for prescribed daily dose at accrual (dQ1 as baseline)**				
dQ1: 1–35 Mg	1.00		1.00	
dQ2: (35–50] mg	1.16	0.92–1.46	1.60	1.02–2.51; *P* = .040
dQ3 (50–70] mg	0.92	0.72–1.18	1.81	1.16–2.82; *P* = .009
dQ4: (70–90] mg	1.07	0.83–1.38	2.01	1.28–3.15; *P* = .003
dQ5: >90 mg	1.40	1.10–1.77; *P* = .005	3.45	2.27–5.23; *P* < .001
Now including interaction for sex by current age group on 3 degrees of freedom (df)				
Interaction regression, 3df	***χ^2 9.43; P = .024***		***χ^2 5.19; P = .158***	
**Sex (baseline: male)**				
Female	0.63	0.45–0.87; *P* = .005	0.90	0.54–1.50
**Current age group (baseline: 25–34 y)**				
<25 y	0.96	0.45–2.06	0.89	0.22–3.70
25–34 y	1.00		1.00	
35–44 y	1.11	0.89–1.38	1.16	0.80–1.69
45+ y	1.60	1.26–2.04; *P* < .001	2.11	1.42–3.13; *P* < .001
**Interaction between female & current age group (baseline: Female & 25–34 y)**				
<25 y	0.72	0.20–2.57	0.44	0.04–5.16
25–34 y	1.00		1.00	
35–44 y	1.67	1.11–2.50; *P* = .013	1.86	1.00–3.43; *P* = .049
45+ y	1.78	1.13–2.81; *P* = .012	1.65	.85–3.23
**Quintiles for prescribed daily dose at accrual (dQ1 as baseline)**				
dQ1: [1–34.5] mg	1.00		1.00	
dQ2: (34.5–50] mg	1.16	0.92–1.46	1.60	1.02–2.51; *P* = .040
dQ3 (50–70] mg	0.93	0.72–1.19	1.81	1.16–2.82; *P* = .009
dQ4: (70–90] mg	1.07	0.83–1.37	2.00	1.27–3.13; *P* = .003
dQ5: >90 mg	1.39	1.10–1.76; *P* = .006	3.44	2.27–5.22; *P* < .001

CI, confidence interval; DRD, drug-related death; HR, hazard ratio

TABLE A6 Proportional hazards regression for methadone-specific DRDs: 16 350 clients in Scotland's 2009–2015 Community Health Index (CHI)-identified methadone-prescription cohort with actual or recovered daily dose of methadone at first CHI-identified methadone prescription and cohort entry in July to December 2009; 102 566 py of follow up

Quintiles for actual or recovered daily dose of methadone at 1st CHI-identified prescription (dQ)<br>dQ regression χ^2 of 49.31 on 4 degrees of freedom; *P* < .00001		
**Events**	**295 methadone-specific DRDs**	
**Covariates [clients, %]**	HR	95% CI for HR;<br>*P*-value *vs* baseline
**Prescription source (baseline: GP prescriber)**		
Other [3036, 19%]	**1.00**	0.74–1.35; *P* = .995
**Sex (baseline: male)**		
Female [5502, 34%]	**1.28**	1.01–1.63; *P* = .040
**Age group at accrual (baseline: 25–34 y)**		
<25 y [796, 5%]	**0.29**	0.09–0.93; *P* = .037
25–34 y [7105, 43%]	**1.00**	**Baseline**
35–44 y [6717, 41%]	**1.71**	1.32–2.23; *P* < .001
45+ y [1732, 11%]	**2.59**	1.84–3.65; *P* < .001
**Quintiles for prescribed quantity at accrual (qQ1 as baseline)**		
dQ1: [1–34.5] mg [2608, 16%]	**1.00**	**Baseline**
dQ2: (34.5–50] mg [3394, 21%]	**1.80**	1.07–3.04; *P* = .028
dQ3 (50–70] mg [3768, 23%]	**2.18**	1.32–3.61; *P* = .002
dQ4: (70–90] mg [3253, 20%]	**2.45**	1.48–4.06; *P* = .001
dQ5: >90 mg [3327, 20%]	**4.03**	2.50–6.52; *P* < .001

CI, confidence interval; DRD, drug-related death; HR, hazard ratio

TABLE A7 Proportional hazards regression for methadone-specific DRDs: 10 183 clients Scotland's 2009–2015 Community Health Index (CHI)-identified methadone-prescription cohort with actual or recovered daily dose of methadone at first CHI-identified methadone prescription and cohort entry after 2009; 42 131 py of follow up

Quintiles for actual or recovered daily dose of methadone at 1st CHI-identified prescription (dQ)<br>dQ regression χ^2 of 16.20 on 4 degrees of freedom; *P* = .0028		
**Events**	**125 methadone-specific DRDs**	
**Covariates [clients, %]**	HR	95% CI for HR;<br>*P*-value *vs* baseline
**Prescription-source (baseline: GP-prescriber)**		
Other [4313, 42%]	**1.80**	1.25–2.61; *P* = .002
**Sex (baseline: male)**		
Female [3242, 32%]	**1.35**	0.93–1.96; *P* = .116
**Age group at accrual (baseline: 25–34 y)**		
<25 y [1195, 12%]	**1.24**	0.60–2.54; *P* = .564
25–34 y [4462, 44%]	**1.00**	**Baseline**
35–44 y [3337, 33%]	**2.92**	1.88–4.54; *P* < .001
45+ y [1189, 12%]	**4.97**	2.92–8.44; *P* < .001
**Quintiles for prescribed quantity at accrual (qQ1 as baseline)**		
dQ1: [1–34.5] mg [2699, 27%]	**1.00**	**Baseline**
dQ2: (34.5–50] mg [2893, 28%]	**0.97**	0.54–1.74; *P* = .910
dQ3 (50–70] mg [2124, 21%]	**1.11**	0.61–2.02; *P* = .733
dQ4: (70–90] mg [1392, 14%]	**2.08**	1.17–3.68; *P* = .012
dQ5: >90 mg [1075, 11%]	**2.25**	1.25–4.05; *P* = .007

CI, confidence interval; DRD, drug-related death; HR, hazard ratio
